# Paradigm shift in medical education due to the COVID-19 pandemic: guidelines for developing a blended learning curriculum in medical education

**DOI:** 10.12688/f1000research.74779.1

**Published:** 2022-01-12

**Authors:** Aisha Rafi, Muhammad Idrees Anwar, Ayesha Younas, Shamaila Manzoor

**Affiliations:** 1 Department of Anatomy, Shifa College, Islamabad, 051, Pakistan; 2 Department of Surgery, Rawalpindi Medical University, Rawalpindi, Pakistan; 3 Department of Medical and Dental Education, Shifa College, Islamabad, 051, Pakistan; 4 Department of Medical Education, Azad Jammu & Kashmir Medical College, Muzaffarabad, Pakistan

**Keywords:** Blended learning, curriculum, guidelines, paradigm shift, Covid-19, Nominal group technique

## Abstract

**Background: **The coronavirus disease 2019 (COVID-19) pandemic has transformed the world’s economy, health and education in a blink of an eye. Almost 1 billion learners have been affected across the globe. This has resulted in a paradigm shift to blended learning. Therefore, it was felt necessary to provide practical guidelines for the development of blended curriculum in medical education. It would help to overcome the challenges faced due to unprecedented transformation of medical education on account of pandemic.

**Methods:** Guidelines based on personal experience of the authors and literature search were developed using the complex adapted blended learning system (CALBS) framework. Seven experts developed these guidelines using the nominal group technique. The consent form and literature for CABLS framework was shared with experts. The experts developed the guidelines independently during phase one of the technique. After a given time, phase 2 started with moderator mediated discussion about the individual guidelines developed by the experts. After discussion and mutual consensus four types of guideline ideas were developed. During the third phase the experts ranked the guideline ideas on a scale of 1 to 5. The guideline idea that ranked highest was selected as a final guideline for developing a blended learning curriculum.

**Results:** The group consensus resulted in developing seven guidelines for a blended course or curriculum in medical education. These guidelines are based on a conceptual framework supplemented by expert’s own personal experience and current evidence from literature.

**Conclusions:** These guidelines would provide a comprehensive and systematic approach to develop a blended learning curriculum in medical education.

## Introduction

Blended learning can be classified as an integrated learning approach where two or more teaching and learning strategies are blended to achieve the learning outcome. Combining modes of web-based technology, pedagogical approaches, instructional technologies with tasks concerned with face-to-face teaching and learning constitute all forms of blended learning. ^1^

Blended learning strategies have been studied in the past with studies concluding that they facilitated an improvement in students’ academic performance, motivation, and attitude along with convenient and flexible learning. ^2^

The coronavirus disease 2019 (COVID-19) pandemic has transformed the world in a blink of an eye in the context of the economy, health and education at all levels. ^3^ Educational landscapes across the globe have seen rapid improvisations at all levels due to partial or complete lock down and other protective strategies to minimize the spread of the virus. ^4^ Almost 1.5 billion learners have been affected worldwide due to pandemic, which ^5^ has resulted in varied responses to education, from complete cessation of all educational activities to a holistic shift to online learning. This has instigated a paradigm shift from traditional face to face or physical classrooms setting to virtual classrooms, which has been challenging for faculty, students and the institutions alike. ^6^ Education in the health professions was not spared this paradigm shift. Realistically speaking, virtual environments traditionally do not cater to the needs of medical education because medical skills can never be achieved exclusively online. ^7^ The pandemic has caused moderate to extreme level of anxiety particularly among the university students. ^8^

At the same time, the COVID-19 pandemic has helped to develop technologically smart medical teachers and students to meet the challenges of digital world. Digital technology and artificial intelligence are the future of medical science and healthcare. This calls for a change in the learning outcomes, expected competencies and entrustable professional activities (EPA) in medical education. ^9^ Virtual reality teaching is a new norm and calls for unprecedented transformation of medical education and the health care system as a whole. One day it will likely become a standard and the term blended will not be used anymore. Very soon blended learning will be a default model. ^10^

**Figure 1.**
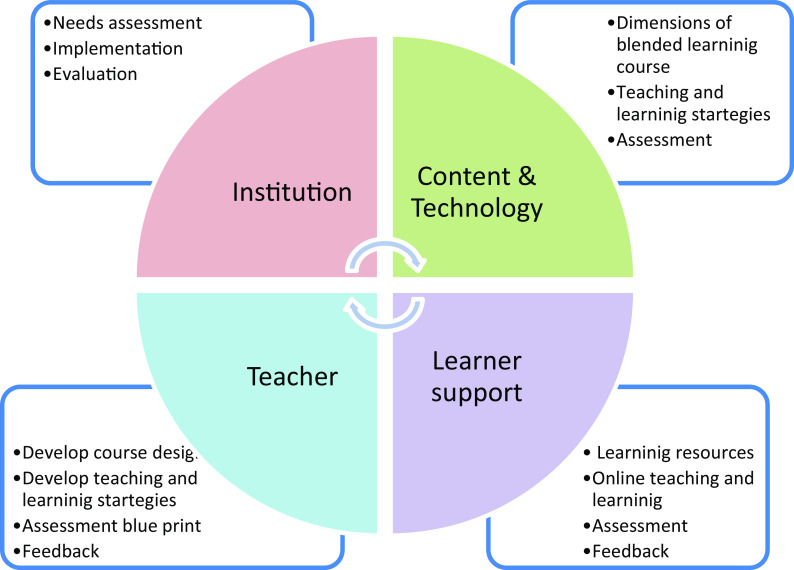
Guidelines for blended learning based on the complex adapted blended learning system (CABLS) framework.

Blended learning programs can use different models and frameworks that best match their outcomes. Planning and implementing a blended learning course entails that we should first understand the related hypotheses and the theoretical underpinnings of the blended learning concept. In medical education the most successful model of blended learning is the rotational model. ^11^ To aid medical educators involved in developing blended learning courses, we developed a set of guidelines utilizing the theoretical framework, complex adaptive blended learning system (CABLS). The main framework is organized around the learner support, the teacher, the content and technology and the institution ( Figure 1). The salient features of the framework are that the learner’s role changes from a passive to an active learner. The learner support including self-regulation, collaborative skills and technical skills etc., to achieve the learning outcome. The teacher must adopt the pedagogical approaches that engage the learners in a blended classroom. The content should be delivered in a blended learning strategy.

The leadership support includes technology, infrastructure, budget, resources, addressing the barriers etc.

## Methods

The present study was conducted in March 2021. The present study was based on personal experiences of the experts of the study supplemented by the literature review, therefore it did not require ethical approval from ethical review board. The CABLS framework for blended learning was used for developing the guidelines proposed in our study.

We utilized an iterative process of nominal group technique (NGT), because it is a suitable technique for developing guidelines besides being cost effective and less time consuming. ^12^ For our study, participants were selected by purposive sampling technique. At least seven participants are recommended for NGT. ^13^ The NGT was modified according to prevailing circumstances due to COVID-19. The online meetings were scheduled for NGT. Medical experts were invited specifically from those universities where blended learning curricula are being implemented in true letter and spirit in lieu of pandemic. Ten proposed experts who fulfilled the inclusion criteria were contacted initially via email. The criteria for selection of the participants were based on their expertise, academic and community practice. The demographic details of experts are given in Table 1. Apart from this it was also ensured that participants were actively involved in developing undergraduate medical and dental curricula and consented to take part in all rounds of NGT. Those who did not consented for full round of NGT were excluded. Faculty members who were not actively involved in curriculum development were also excluded.

**Table 1.** Demographic details of the experts.

S. No	Gender	Age (years)	Qualifications	City
1.	F	62	FCPS (Physiology), FAIMER FEllow	Islamabad
2.	F	46	FCPS (Anatomy), MHPE (CPSP)	Taxila
3.	F	48	FCSP (Surgery),MHPE	Lahore
4.	M	50	PhD, FDS (Orth) RCS, MOrthRCS, FDSRCS, FFDRCSI, BDS MBBS	Islamabad
5.	F	55	MHPE, PhD (Scholar)	Rawalpindi
6.	F	56	MBA, MHPE, PhD Scholar	Rawalpindi
7.	F	46	MHPE	Multan

The details of the proposed study were sent via e-mail along with a consent forms and instructions for the nominal group technique to all the experts. The link for Pertinent literature about the CABLS framework and literature on blended learning was also sent in instructions. The due date for submission of draft was also mentioned. Participants were requested to confirm their availability for all four rounds of the study to ensure reliability of the process. Only six experts acknowledged and consented to be a part of the whole study. The seventh expert was approached face to face in our own institution. As per instructions during silent or phase one of the nominal group technique the experts prepared the initial draft based on the CABLS framework in private. They were instructed to submit the draft via postal address to the principal author within a due date mentioned in instructions. The experts submitted their ideas for guidelines to the principal author after two weeks.

The phase-II was scheduled online on Zoom. The meeting was moderated by the principal author. All the guidelines submitted previously to the principal author was shared on zoom one after the other anonymously. The name of the expert was not shared to control bias. All the experts went through all the guidelines. Before discussion the guidelines that overlap in meaning were combined to develop a new guideline that best conveys the intention of all the combined guidelines.

The moderator presented the guidelines again developed after deleting the duplication and after combining the similar guidelines. This was the longest and crucial stage for developing guidelines. The experts discussed in detail the inclusion of tip or idea for a blended learning course with reference to Covid-19. Some of the experts were of the view that Harden’s ^14^ ten points for developing the curriculum fit in every situation, others did not agree. One of the experts advocated the guidelines given by Kern ^15^ for developing the curriculum. The discussion lasted for two hours. The feasibility was also addressed. After the consensus of the experts four sets of guidelines were developed for the third phase. The two sets of guidelines given by Harden and Kern consisting of consisting of ten steps approach and six steps approach respectively were kept as such. Whereas the other two sets with six and seven guidelines were developed after discussion among the experts. There were ten, seven, six and six guidelines in sets number one (Harden), two, three and four (Kern) respectively ( Table 2). The experts award scores ranging from 1 to 5 to each guideline in the given sets. The moderator add the total points for each guideline ( Table 2). The whole session was recorded. The four sets of guidelines were emailed to the experts for further evaluation. During phase-III the sets of guidelines were ranked from 1 to 5 by the experts. The Zoom meeting was scheduled for the third phase after confirming the availability of the experts. During this phase the four sets of guidelines were ranked from 1 to 5. Number 1 being strongly disagree and number 5 being strongly agreed. Rank 2,3, 4 is interpreted as disagree, neutral and agree respectively ( Table 3). The moderator facilitated the session by sharing the sets of guidelines on the screen and awarding the scores written in the chat box by the experts. The ranked score were added. The set no 2 was ranked highest ( Table 3). Set number 2 consisted of 7 tips or guidelines for developing a blended learning course in medical education. Set number 2 was selected unanimously by all the experts for developing the guidelines ( Table 3). The threat to reliability and validity were controlled during data collection and analysis by anonymity, multiple rounds and controlled feedback. It decreased the social and cognitive biases.

**Table 2.** Scoring by experts for each set of guidelines.

	Scores from each expert for each tip							Frequency of voting	Total score
	#1	#2	#3	#4	#5	#6	#7		
**Set 1**									
Mission and vision	3	1	2	1	1	1	1	7	10*
Needs assessment	5	5	5	5	5	5	5	7	35
Aims and objectives	4	3	3	4	3	3	3	7	23
Content	5	5	5	5	5	5	5	7	35
Organization of content	5	5	5	5	5	4	4	7	33
Educational strategies	1	1	1	1	1	1	2	7	8*
Teaching and learning methods	5	5	5	5	5	5	5	7	35
Method of assessment	5	5	5	5	5	5	5	7	35
communication of curriculum	4	3	3	4	4	5	4	7	27
Management of environment	4	4	4	1	5	4	5	7	27
**Set 2**									
Need assessment	5	5	5	5	5	5	5	7	35
Outline of course	5	5	5	5	5	5	5	7	35
Teaching and learning strategies	5	5	5	5	5	5	5	7	35
Effective resources	5	5	5	5	5	5	5	7	35
Assessment	5	5	5	5	5	5	5	7	35
Communication and implementation	4	4	5	4	4	4	5	7	30
Evaluation	5	5	5	5	5	5	5	7	35
**Set 3**									
Goals	2	3	2	3	1	2	3	7	15*
Aims and objectives	2	3	3	4	2	2	2	7	18*
Teaching and learning strategies	5	5	5	5	5	5	5	7	35
Assessment	5	5	5	5	5	5	5	7	35
Tools for blended learning	4	4	4	4	5	5	5	7	31
Evaluation	5	5	5	5	5	5	5	7	35
**Set 4**									
Problem identification and general Needs assessment	1	0	1	0	0	1	1	7	4*
Targeted need assessment	4	4	4	5	4	4	4	7	29
Goals and objectives	2	2	3	2	2	3	3	7	17*
Educational strategies	5	5	5	5	5	5	5	7	35
Implementation	5	5	5	5	5	5	5	7	35
Evaluation of program	5	5	5	5	5	5	5	7	35

**Table 3.** Ranking of guidelines developed after phase 3 of nominal group technique.

Guidelines #	Expert 1	Expert 2	Expert 3	Expert 4	Expert 5	Expert 6	Expert 7	Total
Set 1	1	1	3	2	4	2	3	16
Set 2	4	4	3	5	4	5	4	29 *
Set 3	2	3	2	1	2	1	1	12
Set 4	1	2	1	2	1	1	2	10
Total	8	10	9	10	11	9	10	67

* Low score.

## Results

The guidelines were developed after thorough discussion during phase II of nominal group technique. All the experts were qualified medical educationists belonging to clinical and basic medical sciences. These guidelines for blended learning curriculum were organized around CABLS framework. Almost all the experts developed guidelines outlining the four basic components of curriculum i.e. outcomes, teaching and learning strategies, assessment and evaluation. Only twenty percent experts wanted to include Mission and vision of the curriculum. The guidelines regarding the mission and vision scored least ( Table 2) because most of the experts were agreed that in context of covid-19 the vision and mission of the curriculum would be same all around the globe. Similarly, the student support and facilities, an important component of CABLS framework was included in guideline for resources. The guideline for educational strategy also scored less ( Table 2). It was discussed that SPICES model given by Harden ^14^ as an educational strategy is for exclusive for on campus delivery of curriculum in medicine. It cannot be applied to blended learning because the social and emotional component in blended learning is not same as in full on-campus curriculum in medicine. The problem identification and general needs assessment guideline given by Kern scored lowest because it was merged into need assessment. Usually the curriculum development began with the goals, aims and objectives, The separate guideline for goals, aims and objectives scored low ( Table 2). The experts had consensus in merging the aims and objectives in the guideline for developing the content for blended learning because they opined that content should always align with the aims and objectives of the curriculum so it should be placed in the same guideline. One of the greatest drawbacks pointed out by all the experts was lack of teaching psychomotor skills online. They were of the opinion that simulations cannot be substituted for real world learning. All the experts stressed upon a face to face component of blended learning in medicine ensuring all the standard operating procedures (SOP).

The set 2 of guidelines scored highest among all the sets of guidelines ( Table 2). It was also ranked highest among the four sets of guidelines ( Table 3). The guidelines given in set 2 are described below:

### Guideline 1: Needs assessment

Needs assessment is a process that seeks need for change to cope with meeting the demands of society at large. ^16^ Qualitative and comparative methods should be used to determine priorities for the most effective use of resources for the blended learning. ^16^

The simplest method of needs assessment is that teachers, clinicians, educationists and students should have several meetings for selecting the course content and mode for blended learning sessions. Blended learning does not mean that the course content should be compromised in any way. The teaching and learning activities should be according to mode of blended learning environment. The effective course design always begins with the needs assessment about the level and type of students who will benefit the program. Assess the learning style of students and their knowledge about the use of digital technology. All the activities, materials, and assignments should support student learning to gain specific outcomes. Assess the faculty needs by what do they want students be able to do and whether they are able to use digital media effectively. The questions to ask are: What is the standard of online sessions? How can students be engaged and assessed online? Can the outcomes be achieved successfully?

### Guideline 2: Develop a course outline

The convergence of two archetypal learning environments calls for systematic integration and alignment between the components in a meaningful way both pedagogically and didactically. ^17^ The content should be chosen wisely so that it is best for each mode of delivery but the two learning environments should be connected in a way to provide a sense of class cohesion and community. ^18^

There should be more learner independence and autonomy in blended learning course design because learner autonomy and self-direction are essential to successful blended courses. The students’ diverse abilities and learning styles should be acknowledged for a successful design. ^19^

The appropriate adjustments should be made according to learner needs and situations i.e. the level of instruction and time allocation to online and face to face learning. Flexibility of the schedule, role of the teacher and student in online and face to face teaching and learning are other components to consider in student support.

A course outline should be developed regarding time allocation, activities, assignments and assessments. Alignment between the components of curriculum helps to determine when, where, and how the outcomes will be achieved. Frequent weekly meetings are required among all the stake holders to decide the dimensions of the program. The frequency of meeting times should be defined by the course structure. ^20^ Every precaution should be observed so that online components should not turn into extended homework in a blended course design. ^18^ Integrate the components in such a way so the students are more motivated to participate and take onus of their learning.

Like in any other course the needs assessment should be transformed into well written measurable objectives. The identified learning needs are linked to formulation of learning outcomes defining the three domains of knowledge, skills, and attitude. ^21^ Great care should be taken in segregating the objectives for different modes of teaching and learning in a blended learning approach.

The curriculum planners should sit together and divide the objectives suitable for online and face to face teaching based on its inherent need, effectiveness, and suitability. This is the key step for blended teaching. The planner must be aware that not all objectives can be achieved either by online and face to face teaching alone. Predominantly the cognitive objectives can suitably be adjusted for online sessions but for attitude and psychomotor skills, one has to be more pragmatic. The psychomotor domain presents a challenging task of replicating the hands-on clinical skills in a synchronous environment. However, simple psychomotor skills, for example, examination of nerve and muscle injury can be taught online with equally comparable results. ^22^ Medicine is a practice-based profession and special care must be taken while developing a table of specification (TOS) for practical skills. ^23^ The complex practical skills which need hand eye co-ordination and hands-on practice must be delivered within a classroom setting, with immediate feedback. This is essential for making safe practitioners. The attitude domain can be taught both with face-to-face interaction and online. ^24^ Many soft skills like communication skills involving counseling a patient can be successfully taught in online sessions. However, one must keep this in mind that internalization of attitude requires patient student interaction. ^25^

### Guideline 3: Develop the blended teaching and learning strategies

Interactive online communication and discussion has resulted in improvement in the quality of e-learning among students. ^26^ The technology should be suitable for planning the methodology and content for flexible and effective delivery of concept. The mastery level of students, resource materials and type of content should be kept in mind while selecting an online teaching and learning tool. ^27^ The teaching and learning strategies should align with the learning objectives. All efforts should be exercised to engage the learner with the learning material. The learner can be engaged during online sessions by providing opportunities for online discussion, empowering students for making their own decisions in the activity based learning, promoting joint ownership and decision making among students and teachers. ^28^

Both synchronous and asynchronous strategies should be used. Some of the most frequently used tools are e-lecture (video podcast), ^29^ case studies & flipped classroom, ^30^ online problem based learning (PBL), ^26^ webinar and video-based instructions. ^32^ Google classroom should be used as asynchronous mode of teaching. ^33^

The flipped classroom strategy has been used in many places even before COVID-19. This strategy is liked by educationists and students alike. In a flipped classroom session, the activity involves the mixing up of the short presentations, questions and answers, zoom polls, clickers to reinforce and summarize the content previously asynchronously on Google classroom.

The learner should be engaged with the learning material with a constructive approach. The online constructivist classroom sessions should be based on activity. Use break out rooms or zoom polls to divide class into small groups. This online tool provides collaborative learning via peer interaction and engagement with the task. The facilitator should use the main room periodically to monitor the progress. The task should be highly structured with appropriate length for meaningful engagement with the learning task. ^34^

The discussion boards are another important tool for an interactive classroom. It helps to address the learner’s issues in real time within the supportive environment. The immediate feedback to the learner would help in solving the problem.

Pre-recorded videos have been used for teaching soft skills. ^35^ Videos can either passively present content to learners or actively engage them by asking them to perform in a live online session.

Tailor the learning environment according to different learning needs and contexts. An online academic debate and panel discussion by experts can be a good addition to revision and reinforcement sessions. It gives a good platform for case-based discussions, case study or problem. This is particularly effective for clinical clerkship sessions. ^36^

The effective use of social platforms should be encouraged in developing a sense of classroom community among the students and the teacher. Adding a Twitter badge to the homepage of the course and using hashtags to post relevant information can generate discussion on this important social platform. Similarly, Facebook features can be used for collaborative learning and communication. ^37^

Many studies claim that online and face to face teaching ensures the same outcomes but one has to be skeptical. ^38^^,^^39^ The psychomotor and affective domain can only be learned best in the real-life scenarios. The psychomotor skills are taught, acquired, performed, and lastly learned in the face-to-face real settings. ^40^ Similarly, the knowledge component for attitude change, can be acquired online but performance and experience can only be acquired face to face. ^41^

Students must have adequate exposure of patients to acquire clinical competencies by becoming active participants of the health care team, and also have periods of independent learning in outpatient and inpatient settings. ^42^ Only when students are involved actively with patient care and have the chance to perform, demonstrate, communicate, and empathize will they be able to fully internalize the professional attitude and will therefore be safe to practice. ^43^

All objectives that require face-to-face interaction should be tabulated with specifications along with instructional strategies. Maximum patient encounter and interaction should be used to consolidate online learning sessions.

### Guideline 4: Develop effective resource materials for a new learning landscape.

Teachers also have to be technologically smart and aware of modern pedagogical approaches. The institute has to ensure a robust faculty development program to make teachers up to date with new advances especially with IT skills and software use. The faculty should devise the activities for effective delivery of content online. The information transfer, resource exploration and collaborative knowledge-creation can be achieved by developing study resources using the mass media. Help the learners via digital simulations for experimental laboratory work. Develop enquiry-based activities like problem-based learning in a blended environment. Prepare hyperlinked documents, audio files, narrated slide shows, interactive image maps and simple interactive games for asynchronous classroom activity. ^43^

The course materials have to be modified in a suitable format like pdf and posted on learning management system (LMS), Moodle etc. Use digital technology to develop a one stop shop solution to all students’ queries and issues. ^44^ Access to state-of-the-art research laboratories, databases, and libraries may be required for perusing advanced studies and research base projects. ^45^

### Guideline 5: Develop an assessment blue print

In blended teaching it is imperative that assessment is provided to check the depth of students’ learning. Many techniques that are used in face to face can also be used in blended teaching. Online assessment strategies in blended programs include quizzes and tests, assignments, individual and group projects, participation in discussions, and proctored face-to-face midterm and final exams. All methods have strengths and limitations while preparing and administering the courses. Therefore, online and traditional assessment methods are used complementarily to overcome their respective weaknesses. Assessment techniques have to be developed using the same blueprint outline as in conventional teachings. This assessment grid includes objective, domain assessment tools, weightage, number of items and type of assessment ( Table 4).

**Table 4.** Assessment grid for blended learning.

Objective	Domain (Cognitive, Psychomotor, Attitude)	Instructional strategy	Assessment tool	Number of items	Marks	Type of assessment
						

Some studies have shown that the use of e-assessment raised efficiency and quality of assessments especially in clinical settings. The face-to-face assessment is more reliable, valid and free from cheating and intellectual dishonesty. The cheating during assessment can be controlled via an online proctoring system where the student is monitored by the invigilator online; however, this requires faculty training. ^8^

The goals, standards and criteria should be set and communicated to stakeholders for the online and face to face component of blended learning. Rubrics and criteria for awarding marks and pass/fail should be communicated. Develop a program of assessment for collecting maximum data points for a pass/fail judgment. In case of unstable internet connection the arrangement for remedial sessions of those students who missed the online session should be made and communicated effectively to the students. ^8^

The continuous assessment can be achieved by recognizing the student’s accomplishment in achieving the competency or performance throughout the session.

Use multiple methods of assessment both for online and face to face tests. The formative assessment should be done using Padlet, Socrative etc.

### Guideline 6: Develop a communication and implementation framework

Blended teaching is more student-centered. The students are responsible for their learning due to the flexible nature of the curriculum as well as the use of technology. The teachers’ relations with students change as students have more control and teachers adopt more facilitative roles. ^28^ A central body, such as a curriculum committee should be responsible for communication of content and mode of delivery. The communication to staff and students should be the responsibility of the working group. There should be proper planning for the material to be communicated to the staff and students. The students should be communicated timetables, study guides, case scenarios for online problem based learning (ePBL), guidelines for e-practical lab sessions as well as for online discussion forums, well ahead of time. The faculty should provide resource materials and associated links well in advance to students. Similarly, instructions and guidelines for online assessment should be communicated in time.

The time for online and on campus classes should be communicated for smooth execution of the program. The students should also be provided with the contact details of the faculty.

### Guideline 7: Evaluate for continuous monitoring and improvement

Feedback has been regarded as the best tool for evaluating the ongoing progress of a program. It is ideal to take feedback after every session from both the students and faculty, as this would help in improving the course to achieve the desired objectives. One of the simplest tools for feedback is an online survey. The survey should be developed on the questions meant for evaluation such as students’ interaction and satisfaction, type of e-learning tool and its impact on learning, challenges faced during implementation and suggestions for improvements.

Pre and post-test exercises are another effective tool to assess the progress of a course and achievement of objectives. The course should be evaluated during and after the session. Make necessary changes after consulting all the stakeholders. The results of evaluations should be used for developing an improved contingency plan for emergency situations encountered in the future.

## Discussion

The COVID-19 pandemic had compelled medical educationists around the globe to design new methods for teaching and learning. Seminars have become webinars and video calls, with web conferencing systems like Zoom and Microsoft teams becoming the exclusive mode of communication during this pandemic. ^46^ Online learning in medical education may not guarantee the same level of quality of education as on campus study but despite this fact, education needs to be continued. Blended courses have been developed for a number of specialties other than medicine and dentistry. It was an immediate challenge to implement blended learning on account of lockdown restrictions. A challenge bigger than this was to develop a blended curriculum for undergraduate medical curriculum.

This study was conducted to address the challenge of developing a blended curriculum in undergraduate medical education. For this purpose, a consensus developing technique (nominal group technique) was adapted for designing guidelines for a blended learning curriculum in medical education. In this democratic method all participants have an equal voice in the process and all the responses are bias free due to feedback and discussion. The process can be adapted according to need and focus of study as well as the constraints of study. ^47^ NGT has been used in a wide range of research studies. NGT allows for adaptation and modification without compromising the basic tenets of the process. ^47^ In this study NGT was modified for online development of the guidelines. Adopting the NGT online allows participation from all over the country. It results in generating a rich dataset from diverse faculty members who are involved in developing different types of curricula. Besides providing innovative ideas an online NGT would also help faculty from underdeveloped countries to learn from international faculty with a minimal resource requirement. This can be shown by this study in which there is a plethora of advice from a learned community of experts.

The goals, aims and objectives of the curriculum are almost similar across the globe due to the suspension of on campus face-to-face activity during the COVID-19 pandemic. Countries have come up with different options and solutions depending upon the resources and the burden of disease. They had prioritized and adjusted accordingly. The aims and objectives define the content to be taught so they are included in the guidelines for the course outline in this study. The objectives of the course were part of the content in another study for developing the curriculum. ^48^ The goal of curriculum development during pandemic is to facilitate the designing and implementation of the curriculum to the emerging education challenges during and after the pandemic. ^49^

It has also been stated in one study that needs assessment stage for curriculum development also took into account the aims, goals and objectives of the curriculum in relation to the problem addressed. ^50^ Needs assessment has been prioritized as the first guideline for developing the blended curriculum in this study. It identifies the actual barriers and problems related to planning a curriculum. The needs assessment not only defines the aims and goals but it also delved into resources required for the curriculum implementation. ^51^

Guideline number two and three about blended learning content and teaching and learning strategies is a must for any type of curriculum. All the experts stress upon the careful selection of content and alignment of learning outcomes with teaching and learning strategies. The blended learning environment should be planned in a pedagogically sound manner for face-to-face and online teaching, representing the best attributes of both learning environments. ^52^ The teaching and learning activities should fit in well with the students characteristics, technology quality, online tools and face to face environment. ^53^

The guideline for developing the effective resources called for overcoming the barriers in terms of cost and training of faculty and students in technology enhanced blended learning environment. ^9^ In this study all the parameters integrating technology in a blended environment for medical education were discussed. The experts had consensus on faculty training and students training for using the digital technology for teaching and learning. Instead of developing the teaching and learning resources from scratch the medical institutions should invest in already available digital learning resources. ^54^ It is advisable that same platforms, tools and setting should be used for training the students and faculty. ^9^

While developing the guideline for assessment blue print all the experts were aware of the basic principle that the learning objectives, and teaching and learning strategies should align with formative and summative assessment. ^9^ The experts of this study stressed the effective use of technology by the students and faculty. All the basic principles for making a valid and reliable assessment be ensured as well, they added.

The guideline for implementation called for addressing the challenges and their emerging responses for curriculum success. Every effort should be exercised to deliver the curriculum in a safe environment. The barrier for successful implementation of blended curriculum lies with the technology for information and communication, simulation modeling technology, faculty readiness and acceptance by the students. ^55^

The last guideline for curriculum evaluation mandated that curriculum should be evaluated during and after the course for formative and summative evaluation. ^23^ Apart from this, blended curriculum should be holistically evaluated including the satisfaction of students and faculty. It should include the feedback from all the stakeholders ^9^^,^^39^

The strength of this study is generating a wide range of ideas on a single problem, prioritizing and final selection of ideas through an anonymous democratic method. All the participants of this study had acknowledged that their opinions and suggestions were given importance in developing the guidelines. There had been an equal opportunity to participate. The non-hierarchical participation reduces influential bias and helped in collecting a rich data. Another strength was that all the participants were working from home due to pandemic, so they were available at a flexible time period.

Weaknesses of the nominal group method is that it addresses a single problem., and the small sample size can affect the validity of results as one random vote can alter the overall ranking of the guidelines. ^47^

This study promotes sharing experiences and experimenting with new pedagogies which may assist in adopting the digital technology. This study would also provide the useful guidance for transforming the curriculum for such emergency situations in the future.

## Conclusion

The shifting of on campus medical education to online owing to the COVID-19 pandemic posed a great challenge to institutions and stakeholders in medical education. It requires a comprehensive and systematic approach to address this challenge. Blended learning in medical education is not an ideal strategy to follow but will preserve the academic sessions of students. The practical sessions can be conducted by strict compliance with standard operating procedures on campus. All stakeholders of the system are required to follow the guidelines in true essence to achieve the desired outcomes.

## Data availability

### Underlying data

All data underlying the results are available as part of the article and no additional source data are required.

### Extended data

Zenodo: Paradigm shift in medical education due to the COVID-19 pandemic: guidelines for developing a blended learning curriculum in medical education. https://doi.org/10.5281/zenodo.5802622. ^56^

This project contains the following extended data:

• CONSENT___Instructions_to_experts_.docx
• Draft_Guidelines_prepared_by_experts.docx
• README_file.docx
• Scores_and_ranking_of_guidelines.docx

Data are available under the terms of the Creative Commons Attribution 4.0 International license (CC-BY 4.0).
