# Supplementary material for: Paradigm shift in medical education due to the COVID-19 pandemic: guidelines for developing a blended learning curriculum in medical education
Source: F1000Res. 2022 Oct 7;11:37. Originally published 2022 Jan 12. [Version 2] doi: 10.12688/f1000research.74779.2 (PMC9634138; doi:10.12688/f1000research.74779.2)
Supplement: Peer review history

# Peer review: Reviewer response for version 2

**DOI:** 10.5256/f1000research.139209.r152709

**Published:** 2022-11-03

**Authors:** Noor Akmal Shareela Ismail

**Affiliations:** 1 Universiti Kebangsaan Malaysia, Kuala Lumpur, Malaysia

I have no reservations to allow this manuscript to be indexed.

Is the rationale for developing the new method (or application) clearly explained?

Yes

Is the description of the method technically sound?

Partly

Are the conclusions about the method and its performance adequately supported by the findings presented in the article?

Yes

If any results are presented, are all the source data underlying the results available to ensure full reproducibility?

Yes

Are sufficient details provided to allow replication of the method development and its use by others?

Yes

Reviewer Expertise:

Medical Education

I confirm that I have read this submission and believe that I have an appropriate level of expertise to confirm that it is of an acceptable scientific standard.

# Peer review: Reviewer response for version 1

**DOI:** 10.5256/f1000research.78566.r144319

**Published:** 2022-08-16

**Authors:** Noor Akmal Shareela Ismail

**Affiliations:** 1 Universiti Kebangsaan Malaysia, Kuala Lumpur, Malaysia

Thank you for sharing your write-up regarding the guideline for a blended medical curriculum. I think this manuscript has highlighted the conduct of developing a guideline with several medical educationists to assist other lecturers/administration with a novice experience in medical education. 

 Since I have a medical education background, I am judging the guideline based on my experience. However, the write-up is a formal narrative of what other people have done elsewhere and the novelty is not something the authors can sell. Almost every institution in the world is adapting to a blended curriculum and all have agreed to test the psychomotor (and affective) skills is difficult as compared to cognitive. But, teachers, as well as learners, do adapt. The practicality is much depending on the resources, strengths, and weaknesses of one's institution. 

 Therefore, I would agree this write-up can assist other institutions to embark on a fully blended medical curriculum. Apart from the typo in Fig 1, I have no issue with the narrative throughout the text, except there was a repetition in one of the earlier paragraphs. I would strongly suggest the authors summarize the methodology in a figure so a non-medical education background can digest the wordings better.

Is the rationale for developing the new method (or application) clearly explained?

Yes

Is the description of the method technically sound?

Partly

Are the conclusions about the method and its performance adequately supported by the findings presented in the article?

Yes

If any results are presented, are all the source data underlying the results available to ensure full reproducibility?

Yes

Are sufficient details provided to allow replication of the method development and its use by others?

Yes

Reviewer Expertise:

Medical Education

I confirm that I have read this submission and believe that I have an appropriate level of expertise to confirm that it is of an acceptable scientific standard, however I have significant reservations, as outlined above.

# Peer review: Reviewer response for version 1

**DOI:** 10.5256/f1000research.78566.r126341

**Published:** 2022-04-06

**Authors:** Naushaba Sadiq

**Affiliations:** 1 National University of Medical Sciences (NUMS), Rawalpindi, Pakistan

The article is written to address the global issue of the closure of medical schools resulting from the COVID-19 pandemic. The guideline will be helpful in establishing a blended learning program.

**Summary of Article;**

 As a result of the challenges faced, the author has conducted this study to develop a guideline with the help of seven experts and nominal group technique. In phase one experts worked independently and developed guidelines. In phase 2, after discussion among the experts in the presence of a moderator, 2 sets of guidelines were developed and 2 pre-existing sets of guidelines for curriculum development available in the literature were considered for the next phase of this study.   During the third phase, the set of guidelines that ranked highest on a scale of 1-5 was selected as a final guideline for developing a blended learning curriculum. This guideline has 7 items/tips for developing a blended curriculum. 

**Review report;**

1. Is the rationale for developing the new method (or application) clearly explained?
**Response; Partly **
2. Are sufficient details provided to allow replication of the method development and its use by others
**Response; Partly **

**Justification of the above two points;**

 In methodology, some terminologies are used interchangeably which can cause confusion in the mind of the reader. 

 for example guideline ideas, guidelines, set of guidelines, and in the end seven guidelines. The statement *"There were ten, seven, six and six guidelines in sets number one (Harden), two, three and four (Kern) respectively ( Table 2)"* is not clearly understood.

 It is suggested that in phase one guidelines were developed by experts independently. In phase two, two sets of guidelines were developed and 2 sets were adopted from literature. In phase three, 4 sets of guidelines were considered. The terminology of " Number of items or tips  " in each set of guidelines can be used to give the details of each guideline.  

 As mentioned above, amendment is required in the statement mentioning *" After the consensus of the experts four sets of guidelines were developed for the third phase"* ****Two sets of guidelines (Harden and Kern)were included in the study from the literature.

 Please give reference to the statement mentioned in the result section " *It was discussed that SPICES model given by Harden ^14^ as an educational strategy is for exclusive for on campus delivery of curriculum in medicine. It cannot be applied to blended learning because the social and emotional component in blended learning is not same as in full on-campus curriculum in medicine.*"

 It is stated that " *we developed a set of guidelines utilizing the theoretical framework, complex adaptive blended learning system (CABLS). The main framework is organized around the learner support, the teacher, the content and technology and the institution ( Figure 1)* **.**" 

 The actual CABLS has six elements that are not overlapping, in this study five elements are used with repetition of items i.e. Assessment. 

 In the section of Develop a course outline  " *Alignment between the components of curriculum helps to determine when, where, and how the outcomes will be achieved.* **"**

 In the final guideline, the tip/item on outcome is not included. In my experience, it is a very important point that must be included in any guideline.

Is the rationale for developing the new method (or application) clearly explained?

Yes

Is the description of the method technically sound?

Partly

Are the conclusions about the method and its performance adequately supported by the findings presented in the article?

Yes

If any results are presented, are all the source data underlying the results available to ensure full reproducibility?

Yes

Are sufficient details provided to allow replication of the method development and its use by others?

Partly

Reviewer Expertise:

Educational research .

I confirm that I have read this submission and believe that I have an appropriate level of expertise to confirm that it is of an acceptable scientific standard.
